# Boosting in the age of Omicron

**DOI:** 10.7554/eLife.87358

**Published:** 2023-04-17

**Authors:** Alex Sigal

**Affiliations:** 1 Africa Health Research Institute Durban South Africa

**Keywords:** COVID-19, vaccine, humoral immunity, Human

## Abstract

A third dose of the inactivated vaccine CoronaVac fails to stimulate the production of neutralizing antibodies which target the Omicron variant.

**Related research article** Zhang H, Hua Q, Xu NN, Zhang X, Chen B, Ma X, Hu J, Chen Z, Yu P, Lei H, Wang S, Ding L, Fu J, Liao Y, Yang J, Jiang J, Lv H. 2023. Evaluation of antibody kinetics and durability in healthy individuals vaccinated with inactivated COVID-19 vaccine (CoronaVac): a cross-sectional and cohort study in Zhejiang, China. *eLife* **12**:e84056. doi: 10.7554/eLife.84056.

Nowadays, most people who get sick with COVID-19 will have a stuffy or runny nose, a sore throat, a cough and a headache, sometimes accompanied by fatigue or mental fog. While unpleasant, these symptoms are not as dangerous as lung damage and breathing difficulties, which were common earlier in the pandemic. The inflection point took place in late 2021 and early 2022, closely linked to the emergence of Omicron subvariants which infect the upper parts of the respiratory tract better than the lower regions, mostly sparing severe infection of the lung. In conjunction, people gained extensive immunity through vaccination, previous infections, or a combination of both (Sigal, 2022).

Neutralizing antibodies, which are designed to block a specific virus from entering cells, are a major component of this immunity. COVID-19 vaccines aim to make the immune system produce these molecules, with the level of neutralizing antibodies released after immunization being strongly correlated with vaccine efficacy against symptomatic disease (Khoury et al., 2021). However, the amount of these protective antibodies decreases over time and additional ‘booster doses’, which complement the initial two-dose vaccination course, have been the main strategy used to counter this waning immunity. Yet Omicron subvariants have also evolved to evade neutralizing antibodies that could successfully deactivate earlier versions of SARS-CoV-2 (Cele et al., 2022).

Most current COVID-19 vaccines are designed based on one of several platforms; these include mRNA technology (as for the BNT162b2 vaccine by Pfizer), or using a whole inactivated virus (like CoronaVac, from the Chinese company Sinovac Biotech). Nations can either make their own vaccines or import them from abroad, and a variety of plausible reasons exist in favor of local manufacture, from national prestige to lower costs. Dependence on import can also be challenging if trading partners hold back doses to prioritize their own populations, or if the product poorly matches local needs, for example by requiring frozen storage. However, considering which type of vaccine works best should be an important consideration at this stage of the pandemic.

CoronaVac is currently used extensively in China, and it is also widely exported to countries such as Indonesia, Brazil, Pakistan or Turkey (Mallapaty, 2021). The consensus is that two CoronaVac doses prevent about 50% of vaccinees from getting sick, with a higher protection against severe disease that is maintained even against Omicron. Vaccine effectiveness against Omicron, however, is only about 25% for mild or moderate disease in people aged 20–59 (McMenamin et al., 2022; World Health Organization, 2021). This decrease in protection matches results showing that in a group of 30 individuals triple vaccinated with CoronaVac, only one person produced a neutralizing antibody response above the detection limit against Omicron (Cheng et al., 2022). More work is thus needed to confirm these findings, and to better characterise the immune response triggered by CoronaVac. Now, in eLife, Jianmin Jiang, Huakun Lv and colleagues – including Hangjie Zhang and Qianhui Hua as joint first authors – report that a third CoronaVac dose elicits neutralizing antibodies against the original strain of SARS-CoV-2, but not against Omicron (Zhang et al., 2023).

The team (who are based at various Centers for Disease Control and Prevention across China, Ningbo University, and Xiamen University) tracked neutralizing antibody responses in volunteers from the Zhejiang Province. They examined the immune response of over 1,000 individuals who had received one or two doses of CoronaVac, while also monitoring antibody production in 90 adults who received three CoronaVac injections during the study period.

Zhang et al. found that antibody responses had waned six months after second vaccination, and that it had become undetectable in most vaccinated individuals after a year. A third injection substantially increased the levels of neutralizing antibodies against an ancestral strain of SARS-CoV-2, as well as the Delta variant. However, Omicron neutralization remained low even after the third dose. Boosting with CoronaVac may therefore still protect against severe disease, but these results suggest that it is unlikely to play much of a protective role against the current dominant variants, at least through neutralizing antibody immunity. While this was not tested by Zhang et al., other work suggests that using the mRNA vaccine BNT162b2 as a booster after an initial course of CoronaVac may elicit a much better production of neutralizing antibodies against Omicron (Cheng et al., 2022).

The findings of Zhang et al. have implications for how to control SARS-CoV-2, but also Pathogen X, the hypothetical virus which will lead to the next pandemic. This work adds to existing evidence showing the strengths and weaknesses of inactivated virus vaccines such as CoronaVac, and how these can be combined with other vaccine platforms to get the best results.
